# Visualizing pathology: The development of a narrated video autopsy for medical students

**DOI:** 10.1016/j.acpath.2026.100241

**Published:** 2026-03-18

**Authors:** Constance Wraith, Michael Osborn, Karim Meeran, Amir H. Sam

**Affiliations:** a Histopathology Specialty Trainee and Quality Assurance Teaching Fellow at Imperial College School of Medicine, London, United Kingdom; b Consultant Histopathologist for Northwest London Pathology, London, United Kingdom; c School of Medicine at Imperial College London, London, United Kingdom

**Keywords:** Autopsy, Pathology, Teaching, Undergraduate

## Abstract

Experiencing an autopsy is a valuable educational tool for medical students, but declining autopsy numbers have made it increasingly rare for students to observe one. This report details the development, implementation, and evaluation of a novel, video-based autopsy teaching session at a large Medical School in London. In the session, a pre-recorded, narrated autopsy was shown to fifth-year medical students, along with interactive quizzes. The session, led by an experienced pathologist, aimed to enhance students’ understanding of autopsy procedures. The effectiveness of the session was evaluated using pre- and post-session questionnaires on a Likert scale. Between 84 and 166 participants answered both the pre- and post-surveys for each statement. After the teaching session, significantly more students reported that observing an autopsy was helpful for their learning, they understood why a patient might undergo an autopsy, they knew what takes place during an autopsy, they appreciated why an autopsy might be important in a patient's care and they understood how correlating clinical history to autopsy findings can help clinicians establish a cause of death (P < 0.001). Furthermore, the number of students who would rather attend a video autopsy session than an in-person autopsy, if given the choice, also increased significantly (P < 0.001). The session allows large numbers of students to become more familiar with autopsy practice and its role in patient care during a single timetabled session. As autopsy numbers decline globally, this innovative approach could be adapted for other health professions and educational levels.

## Introduction

In addition to its crucial role in identifying the cause of death of patients for hundreds of years, the autopsy has long been used in medical education as a teaching practice. Medical students acknowledge the importance and applicability of autopsy in their education.^1^ Previous work has found that autopsies can help students understand the pathophysiology of diseases such as heart failure and pulmonary embolism,^2^ as well as enhance their coping mechanisms in dealing with death^3^ and aid students in practicing deductive reasoning and problem solving.^4^ It is also known that minimal exposure to autopsy during undergraduate medical training can lead to a lack of practical understanding of how an autopsy works. This has repercussions for a student's clinical career when they may need to request an autopsy or discuss the process with a bereaved family.^5^

However, the number of students who have the opportunity to observe an autopsy as part of their medical education is declining,^6^ due to a variety of factors. The number of autopsies worldwide is decreasing. There are several reasons for this, including attitudes of clinicians and families, advancements in technology, economic concerns, and fears of legal liability.^5^^,^^7^ Increasingly, medical students and doctors have a limited understanding of autopsy practice.^8^ Medical school curricula are also becoming increasingly busy, with many educators competing for space in the students’ timetable, leading to pathology being slowly dropped from the undergraduate curriculum. The lack of exposure to autopsy practice not only removes a valuable learning opportunity for students but also has knock-on effects on the specialty of pathology.^5^

Despite declining numbers of autopsies taking place, many medical students in the United Kingdom are still given the option to attend; however, these opportunities frequently rely on students contacting mortuaries directly.^9^ Furthermore, the behavior and attitudes of mortuary staff are a key factor in the benefits students can gain from attending and, to have maximum benefit, autopsy teaching should be structured and delivered by an instructor who behaves appropriately.^9^ With students often relying on luck to even have the chance to attend an autopsy, ensuring mortuary staff can teach students in a supported way that also aligns with their specific course learning objectives is even more challenging.

Some work has been undertaken to find solutions to the issue of dwindling access to autopsy for medical students. Many of these projects explore the use of digital technology to provide students with autopsy teaching. One solution that has been proposed is the use of virtual or augmented reality, though there is limited research evaluating this as a learning tool.^10^ Even if proven effective, the use of virtual reality in teaching requires significant investment in equipment, expertise and development time, which may not be feasible at all institutions. To date, there is no autopsy teaching that we are aware of that utilizes a pre-recorded autopsy to teach medical students this important aspect of pathology.

Given this, we developed and ran an innovative video-recorded, narrated autopsy to be run in a lecture-style environment as part of the pathology course at our institution, for fifth-year medical students. The teaching session aimed to increase students' understanding of why a patient might undergo an autopsy, what an autopsy entails, and how autopsy findings can be correlated with clinical history to understand a patient's cause of death. In this study, we describe the creation of the pre-recorded autopsy teaching session and the evaluation we undertook to understand how students perceive this teaching. As far as we are aware, this is the first time autopsy teaching has been taught to medical students in the United Kingdom using this method.

## Materials and methods

### Setting

We incorporated recorded autopsy teaching into the curriculum for fifth-year medical students at Imperial College School of Medicine. The autopsy teaching takes place in a 5-hour-long, timetabled event, where a pre-recorded, narrated autopsy is shown to students. The teaching takes place in a lecture theatre. A pathologist is present in the lecture theatre throughout to add further narration and answer questions from students. Students also participate in interactive online quizzes during the session, answering questions on what they have seen.

### Session development

Our institution already utilizes hands-on cadaveric prosection as a teaching practice for students on the MBBS course. A pathologist who had experience teaching on the pathology course at our institution conducted a limited, hospital autopsy-style examination of the chest and abdomen of a male cadaver donated for use in medical education in the Human Anatomy Unit. The process was narrated in real time by the pathologist and was recorded on video. Permission to undertake and record the autopsy and show it as part of a teaching session was granted from the Human Tissue Authority (HTA), the independent regulator of organizations removing, storing and using human tissue for, among other purposes, education and training and research in the United Kingdom, prior to filming taking place.

The face of the cadaver was kept covered throughout the filming. After the evisceration, the thoracic and abdominal organ systems were also dissected on film. Included in the film were specimen pots (specimen buckets) from our pathology collection. These were used to demonstrate different pathologies that may be found during an autopsy, with an explanation of how pre-mortem symptoms can be correlated with the pathologies seen. The discussion also covers how the autopsy process may differ for a hospital autopsy, versus a forensic autopsy, or for a patient found dead in the community.

**Fig. 1:**
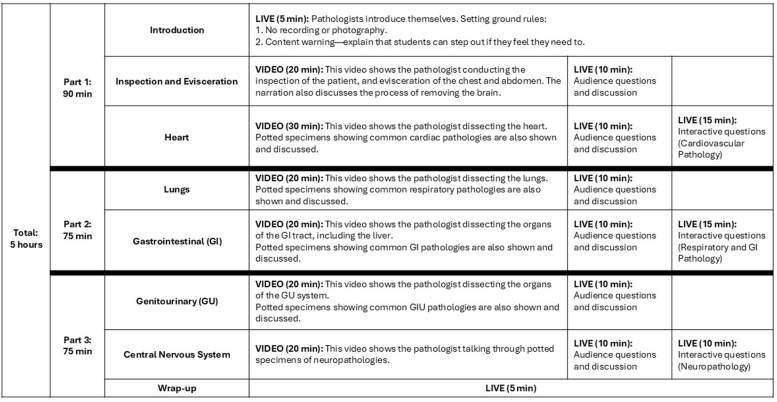
Virtual autopsy teaching session plan including timings.

During the teaching, the recordings were shown in several separate parts of up to 30-minute segments on a screen in a large lecture theatre. In between the films, students were invited to ask questions to the pathologist in the room, as well as take part in interactive quizzes to test their knowledge, hosted on Mentimeter™ (a cloud-based tool). These quizzes consist of questions related to the pathologies seen during the video autopsy and in the potted specimens in the films. For example, ‘give a likely cause of this patient's pleural effusion’. Participation in the quizzes forms part of the students' summative assessment for pathology for the year. The teaching session lasted 5 hours in total, with two breaks [Fig. 1]. In line with permissions from the HTA, the lecture was only shown to students attending in person and was not recorded for students to watch at a future date.

### Session evaluation

The study received ethical approval from the Imperial College London Education Ethics Review Process (EERP 2425-026). The autopsy teaching was presented to students as a mandatory component of the standard teaching provision, although participation in this study was entirely voluntary. All participants provided informed consent before completing the questionnaire.

Participants completed five-point Likert scale pre- and post-session surveys on their mobile device, rating their level of agreement with several statements developed from the intended learning outcomes of the session, from strongly disagree to strongly agree. They were also asked whether they had seen an autopsy before (pre-survey only) and if they would rather receive autopsy teaching in a ‘virtual’ format or in person. The word virtual was used throughout the questionnaire to describe the video autopsy session. Using the same device, participants completed each survey anonymously on Mentimeter™ and pre- and post-intervention responses were automatically matched using the platform's software.

Pre- and post-Likert scale responses were converted into numerical scores between 0 (strongly disagree) and 4 (strongly agree). We analyzed data using IBM SPSS Statistics (Version 28.0, IBM, Armonk, New York). We compared self-reported agreement with each statement in the pre- and post-teaching surveys using the Wilcoxon signed-rank test or the chi-squared test for matched pairs.

## Results

166 out of 203 (82 %) medical students attending the teaching session volunteered to have their anonymised data processed as part of the research. However, not all these students completed all statements on the questionnaire, resulting in a final sample size of between 84 and 166, depending on the statement.

The pre-teaching survey revealed 151 of 166 respondents (91 %) had not previously observed an autopsy, either in person or virtually.

Table 1 shows a comparison of the median, and lower and upper quartile Likert scale scores before and after the teaching, as well as the results of statistical significance testing. A statistically significant difference was seen in response to five out of six statements asked.

**Table 1:** Median Likert scale responses before and after teaching and associated tests of statistical significance.

Statement	Number of respondents	Median score (LQ-UQ) before the teaching	Median score (LQ- UQ) after the teaching	Median Paired Difference (LQ-UQ)	Wilcoxon Signed- Rank statistic
Medical students should be required to attend an autopsy as part of their training.	101	3 (2–4)	3 (2–4)	0 (0–1)	802.5
Observing a virtual autopsy was helpful for my learning.	101	3 (2–3)	3 (3–4)	1 (0–1)	1765.0∗
I understand why a patient might undergo an autopsy.	101	3 (2–4)	4 (3–4)	0 (0–1)	1424.0∗
I know what takes place during an autopsy.	91	1 (0–1)	4 (3–4)	3 (2–3)	4095.0∗
I appreciate why an autopsy might be an important step in a patient's care.	91	3 (2–3)	4 (3–4)	1 (0–1.75)	1489.0∗
I understand how correlating clinical history and autopsy findings can help clinicians establish a cause of death.	91	3 (2–4)	4 (3–4)	1 (0–1.75)	1270.0∗

Scale: 0 = strongly disagree to 4 = strongly agree. LQ = lower quartile, UQ = upper quartile. ∗Statistically significant at P < 0.001.

**Fig. 2:**
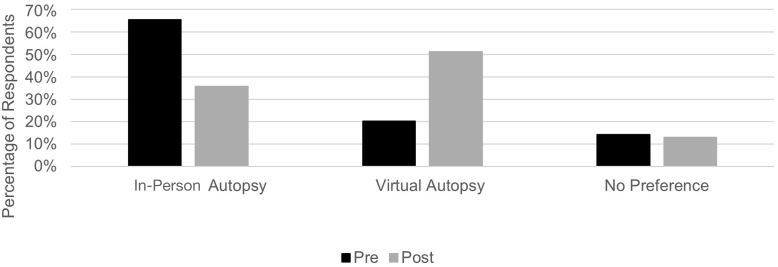
Students' preferences for in-person versus virtual autopsy teaching before and after the teaching session.

Before the teaching, given the choice between an in-person or virtual autopsy, 17 out of 84 (20.2 %) students would rather attend a virtual autopsy. This showed a statistically significant increase after the teaching session to 43 out of 84 (51.2 %) students (*X*^2^ 18.7, P < 0.001) [Fig. 2].

## Discussion

As far as we are aware, this is the first time a recorded, narrated autopsy has been used for pathology teaching at a UK medical school. The session allows a large number of students to become more familiar with autopsy practice and its role in patient care during a single timetabled session. It also provides equitable access, predictability, and structure to the teaching. As a result, students can be appropriately prepared for the teaching, the required learning objectives can be addressed, and appropriate support can be made available for students who may find the teaching emotionally challenging.

Our study also found that students’ preferences for attending in-person vs virtual autopsies changed, with a statistically significant increase in students preferring to attend a virtual autopsy following the session. Videos have been used with great success to teach students in other curriculum areas, including clinical skills and professionalism,^11^^,^^12^ so it is perhaps unsurprising that students consider this a helpful method in autopsy teaching. Whilst we did not collect data on reasons for this change, it could be an area for further qualitative research. We hypothesize possible reasons might include practical ones, such as students being able to sit down and take multiple breaks during the virtual session, which may not be feasible in in-person autopsies. Additionally, a virtual autopsy may feel more detached than an in-person autopsy, as it lacks many of the sensory elements of the mortuary environment that some find off-putting. This could be viewed as a limitation of this teaching method, as these elements are an unavoidable part of autopsy practice that doctors must get used to if they perform autopsies frequently. However, this is less likely to be relevant at an undergraduate level, when the aim is to familiarize students with autopsy practice, or for doctors or health professionals outside pathology who do not perform autopsies as part of their role.

Our study has some limitations. Firstly, the data collection utilized self-reported ratings of understanding, collected immediately after the teaching had taken place. This does mean some caution is required as to whether understanding has objectively increased. Future work could focus on evaluating the effectiveness of the teaching in enhancing student understanding through a formal assessment.

Secondly, this research only reports findings from one cohort at one medical school in the United Kingdom. It is not possible to ascertain the generalizability of these findings to students in different contexts.

As autopsy numbers both in the UK and internationally decrease, accommodating medical students, whose numbers are increasing, will become more challenging. Using a narrated, recorded autopsy allows many more students to view an autopsy than would otherwise be possible. Furthermore, it is more convenient and predictable for undergraduate administrators to schedule this initiative in students’ timetables. While there is some initial resource required to create the video, once it has been made, it requires few further resources to run the teaching session.

We are sharing this work to spark ideas, not just for pathology leads on other undergraduate medical courses, but also for the other healthcare professions. There is also scope for this method of teaching autopsy to be used at a postgraduate level, including in pathology training programs, and for the teaching session to be developed to cover further topics on the undergraduate medical curriculum related to pathology. This could include confirmation of death, death certification, and medical ethics, such as cultural beliefs surrounding death and dying.

Furthermore, future studies could evaluate the effectiveness of teaching using the recorded autopsy. For example, an analysis of students' examination results in pathology examination questions pre- and post-intervention to investigate if the teaching session has an impact on outcomes of assessment in pathology. Additionally, there is scope for qualitative research to explore our findings further. This includes the reasons for the change in students' preferences for in-person versus virtual autopsy teaching before and after the session. Students’ views on the value of observing an autopsy as part of their training could be explored further through focus groups.

## Ethical approval

This study was approved by the Imperial College London Education Ethics Review Process (EERP 2425-026).

## Funding

The initial video recording was funded by an Education Grant from the Pathological Society of Great Britain and Northern Ireland.

## Declaration of competing interest

The authors declare the following financial interests/personal relationships which may be considered as potential competing interests:

Michael Osborn reports financial support was provided by Pathological Society of Great Britain and Ireland. Other authors declare that they have no known competing financial interests or personal relationships that could have appeared to influence the work reported in this paper.
